# Role of Leptin in Non-Alcoholic Fatty Liver Disease

**DOI:** 10.3390/biomedicines9070762

**Published:** 2021-06-30

**Authors:** Carlos Jiménez-Cortegana, Alba García-Galey, Malika Tami, Pilar del Pino, Isabel Carmona, Soledad López, Gonzalo Alba, Víctor Sánchez-Margalet

**Affiliations:** 1 Department of Medical Biochemistry and Molecular Biology, School of Medicine, Virgen Macarena University Hospital, University of Seville, 41073 Seville, Spain; cjcortegana@us.es (C.J.-C.); albaggaley97@gmail.com (A.G.-G.); mali_k@hotmail.es (M.T.); slopez9@us.es (S.L.); galbaj@us.es (G.A.); 2 Unit of Digestive Diseases, Virgen Macarena University Hospital, 41073 Seville, Spain; pilardelpino4@gmail.com (P.d.P.); icarmonasoria@gmail.com (I.C.)

**Keywords:** fatty liver, steatohepatitis, obesity, metabolic syndrome, leptin

## Abstract

Non-alcoholic fatty liver disease (NAFLD), which affects about a quarter of the global population, poses a substantial health and economic burden in all countries, yet there is no approved pharmacotherapy to treat this entity, nor well-established strategies for its diagnosis. Its prevalence has been rapidly driven by increased physical inactivity, in addition to excessive calorie intake compared to energy expenditure, affecting both adults and children. The increase in the number of cases, together with the higher morbimortality that this disease entails with respect to the general population, makes NAFLD a serious public health problem. Closely related to the development of this disease, there is a hormone derived from adipocytes, leptin, which is involved in energy homeostasis and lipid metabolism. Numerous studies have verified the relationship between persistent hyperleptinemia and the development of steatosis, fibrinogenesis and liver carcinogenesis. Therefore, further studies of the role of leptin in the NAFLD spectrum could represent an advance in the management of this set of diseases.

## 1. Introduction

Leptin is a 16 kDa adipocyte-derived hormone described for the first time by Zhang et al. (1994) as the product of the *obese (Ob) gene* [1], although its existence was predicted some decades before in leptin-deficient (ob/ob) and leptin receptor-deficient (db/db) mice [2,3]. Leptin primary amino acid *sequences* show differences in vertebrates, while secondary and tertiary structures are similar [4] and alike to the long-chain helical cytokine family, which includes interleukin (IL) 6, IL-11 (interleukin 11), G-CSF (granulocyte-colony stimulating factor) or oncostatin M, among many others [5].

Leptin is characterized by having pleiotropic effects due to the great variety of leptin receptors (known as Ob-R or LEPR), thus being able to affect many biological processes at different levels. The six existing spliced Ob-R forms are called Ob-Ra, Ob-Rb, Ob-Rc, Ob-Rd, Ob-Re and Ob-Rf, and belong to the class I cytokine superfamily [6,7] but differ from each other in the lengths of their cytoplasmic regions [8]. The most important leptin receptor is the long isoform Ob-Rb since it can fully transduce activation signals into the cell [9], including signaling pathways such as Janus kinase (JAK) 2/signal transducer and activator of transcription (STAT) 3, insulin receptor substrate (IRS)/phosphatidylinositol-3 kinase (PI3K), or Src homology 2 domain-containing phosphatase 2 (SHP2)/mitogen-activated protein kinase (MAPK) [10].

This adipokine, leptin, is mostly recognized for playing a key role in the central control of both energy metabolism [11] and obesity [12], but also has important regulatory functions in different physiological systems and diseases, such as reproduction [13], bone physiology [14], autoimmunity [15], and cancer [16], among many others [17]. Moreover, in the last few decades, data from experimental models and both observational and interventional studies have shown that leptin plays a role in non-alcoholic fatty liver disease (NAFLD) [18], a clinicopathologic entity which develops in the absence of excessive alcohol consumption (typically defined as <20 g per day in women and <30 g per day in men) and comprises a spectrum of diseases, that include steatosis, non-alcoholic steatohepatitis (NASH), hepatic fibrosis, cirrhosis, and hepatocellular carcinoma (HCC) [19].

Today, several problems are associated with NAFLD. This entity is the leading cause of liver disease worldwide and its prevalence is increasing [20], affecting both adults and children [21]. Most patients are asymptomatic for a long time, making it difficult to identify and manage NAFLD and its progression and, in most cases, the disease is detected in advanced stages [22,23]. In addition, there is no authorized effective pharmacological treatments to improve patient outcomes [24]. Therefore, the need to increase research efforts on effective diagnostic and prognosis is essential, suggesting leptin is a powerful tool in the disease. For all those reasons, the purpose of this article is to review the existing literature to better understand the role of leptin in the NAFLD spectrum and to take this hormone into account as a possible clinical non-invasive biomarker or target of treatment for this disease.

## 2. Non-Alcoholic Fatty Liver Disease (NAFLD): Characteristics and Signaling Pathways of Leptin Receptor

NAFLD is a clinicopathologic entity comprising a broad spectrum of liver diseases ranging from simple steatosis to NASH, a more aggressive form of NAFLD and associated with varying degrees of hepatic fibrosis, cirrhosis, and HCC [25]. NAFLD has been rapidly driven by daily life, including actions such as sedentarism or excessive caloric intake compared to energy expenditure, affecting about 25% of the world population [19,20,26]. This pathological condition is detected in approximately 90% of obese (body mass index, BMI ≥ 30 kg/m^2^) and 25% of lean patients (BMI 20.0–24.9 kg/m^2^) and can be affected by other factors (e.g., age, sex, and race) [27]. The highest rates of NAFLD are in South America (31%) and the Middle East (32%), followed by Asia (27%), the United States (24%), Europe (23%), and Africa (14%) [28]. This makes NAFLD in the leading cause of liver disease worldwide and will probably become the most common indication for liver transplantation and the most frequent etiology of HCC in the following decades [21,27].

NAFLD is also considered the hepatic component of the metabolic syndrome, whose prevalence is increasing worldwide at the same time as obesity and type 2 diabetes-mellitus (T2DM) [29,30]. In fact, the Latin American Association for the Study of the Liver (ALEH) recommended the renaming of NAFLD to “Metabolic Dysfunction Associated Fatty Liver Disease (MAFLD)”, and the adoption of positive criteria to diagnose the disease, independently of alcohol intake or other liver diseases. By contrast, the American Association for the Study of Liver Diseases (AASLD) required that there is no significant alcohol consumption or coexisting etiologies of chronic liver disease [23,31,32].

The pathogenesis of NAFLD entail a complex interplay between environmental factors, obesity, changes in the microbiota and predisposing genetic variants that result in altered lipid homeostasis and hepatocyte triglyceride accumulation [19]. In turn, NAFLD is mainly related to metabolic syndrome and adipokines, which not only contribute to pathogenesis, but are also involved in the progression to NASH and cirrhosis [30,33]. In this sense, the physiological role of leptin in the liver was known before this adipokine was discovered, as both db/db and ob/ob mice were shown to present alterations in the liver function, including steatosis [34,35]. Table 1 summarizes research articles related to human NAFLD throughout this review.

### Leptin Receptor Signaling and NAFLD

**Figure 1:**
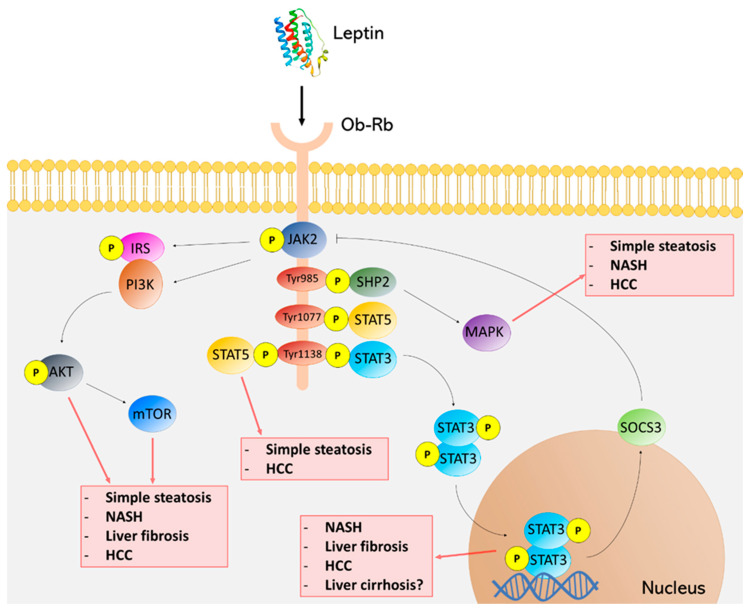
Leptin signaling pathways in the NAFLD spectrum. Leptin/Ob-Rb interaction activates different pathways via JAK2 phosphorylation. The consequent signaling cascade can exert a disruptive role through the activation and phosphorylation of some component implied in this signaling network, such as signal transducer and activator of transcription (STAT)3, STAT5, mitogen-activated protein kinase (MAPK) or AKT/mammalian target of rapamycin (mTOR) pathways, thus favoring some malignancies of the NAFLD spectrum.

Leptin acts by binding to its receptors. Specifically, Ob-Rb isoform is the main leptin receptor as it provokes signaling cascades [31]. After the binding between leptin and Ob-Rb in hepatic cells, intracellular signaling is initiated and JAK2 phosphorylation and activation is allowed. Thus, three tyrosine residues (Tyr985, Tyr1077 and Tyr1138) located in the intracellular domain of Ob-Rb are phosphorylated by JAK2. Tyr985 induces SHP2 signaling pathway and the activation of MAPK, Tyr1077 mediates the activation of STAT5, and Tyr1138 activates both STAT5 and STAT3 [30,44,45,46,47,48]. Subsequently, STAT3 leads to increased gene expression of suppressors of cytokine signaling (SOCS)-3, which acts as a negative feedback inhibiting both leptin and insulin signaling. SOCS-3 overexpression causes resistance to those hormones. Therefore, SOCS-3 downregulation could be a potential approach to prevent and/or treat hepatic diseases [46,49,50]. JAK2 activity is also modulated by phosphorylation of both IRS1 and IRS2, and activation of PI3K, which is essential for leptin to exert its effect on food intake. Likewise, adenosine monophosphate-activated protein kinase (AMPK) activity is stimulated by leptin in peripheral tissues promoting catabolic pathways such as fatty acid oxidation or glucose transport and inhibited in the brain to regulate food intake through a series of hypothalamic neuropeptides [49]. Phosphatidyl inositol 3-Kinase/PI3K/Akt/mammalian target of rapamycin (mTOR) is also activated, improving insulin sensitivity in the liver by suppressing hepatic glucose production [51]. Figure 1 outlines leptin signaling pathways and their implications in the NAFLD spectrum.

Leptin and other inflammatory adipokines such as IL-6 or TNF-α (Tumor nechrosis factor) promote insulin resistance, which has been extensively described in the pathophysiology of NAFLD during the last few decades [52,53,54], as it provokes the inhibition of lipid oxidation together with increased synthesis of fatty acids and triglycerides [19,36,37]. Specifically, leptin could antagonize some insulin functions by modifying the sensitivity of adipocytes to the inhibitory action that insulin exerts on lipid accumulation, decreasing the binding capacity of insulin receptors in the liver, and inhibiting insulin secretion in pancreatic islets [36,37,38]. Hyperleptinemia damages pancreatic β-cells and inhibits JAK2/PI3K signaling in obese patients with T2DM and NAFLD. This signaling pathway is known as the “leptin-insulin pathway” and under normal conditions is activated to regulate glucose metabolism. In addition, hyperleptinemia increases the expression of sterol regulatory element-binding protein 1 (SREBP-1) in the liver, causing lipogenesis [55]. In addition, Sahin-Efe et al. (2018) demonstrated that leptin levels were increased in patients with T2DM, thus being a risk predictor for the development of this disease [56]. 

Moreover, some pathologies can cause NAFLD. This is the case of congenital or acquired lipodystrophy, which is characterized by the total or partial absence of subcutaneous adipose tissue and promotes ectopic accumulation of fat in other locations, including the liver, which leads to severe insulin resistance and the development of NAFLD [57,58]. Patients with lipodystrophy are treated with leptin recombinant treatments, such as metreleptin, approved by the United States Food and Drug Administration (FDA) and the Japanese Pharmaceuticals and Medical Devices Agency, since it improves many associated metabolic disorders such as insulin sensitivity, glucose tolerance, hypertriglyceridemia or NAFLD [59,60,61]. At the same time, work is being done on the development of leptin analogues, leptin receptor agonists or drugs that act on downstream leptin pathways [62].

## 3. Leptin in the NAFLD Spectrum

**Figure 2:**
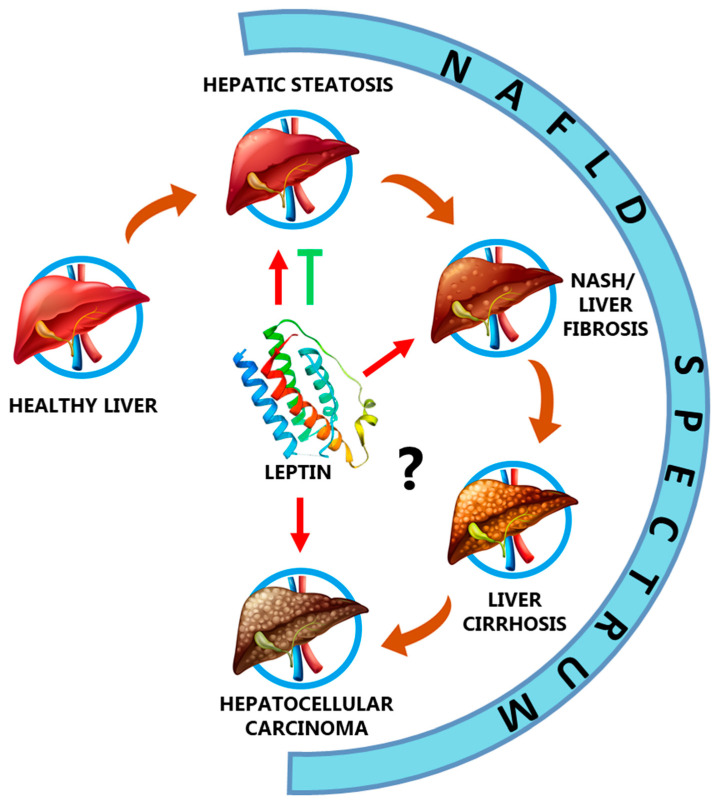
Role of leptin in the NAFLD spectrum. Leptin has been demonstrated to have antisteatotic effects, although this hormone could also contribute to worsening of hepatic steatosis under certain circumstances such as hyperleptinemia. In addition, leptin is involved in the pathogenesis of NAFLD by promoting NASH and liver fibrosis. However, the role of leptin in NAFLD-related cirrhosis and NAFLD-related hepatocellular carcinoma is unknown, but there is much evidence to confirm the protumoral role of this adipokine in liver malignancies Table 1.

NAFLD comprises a set of liver diseases, some of them irreversible. NAFLD development is divided into three main steps: simple steatosis, NASH, and liver cirrhosis. However, NAFLD can eventually trigger in HCC. Simple steatosis is caused by factors such as high-fat and/or high sugar diet, obesity, T2DM, and other metabolic diseases, while NASH can be developed by inflammation and hepatocyte apoptosis. If liver fibrosis is provoked in this step, cirrhosis (and possibly HCC) will be also developed [63]. In the following sections we are going to review the role of leptin in the pathogenesis of NAFLD, which is summarized in Figure 2.

### 3.1. Leptin and Hepatic Steatosis

Hepatic steatosis has different degrees of severity related to liver damage in NAFLD: from simple steatosis to NASH, which is the most important disease in the NAFLD spectrum, since its prevalence is estimated to be approximately 1.5–6.5% in the general population, and considerably increasing this percentage in obese individuals [20]. Although most patients present isolated steatosis, about one third develop NASH, which confers a higher risk of progression to more advanced stages of NAFLD. In this step, inflammation develops when triglycerides levels exceed hepatic physiological adaptive mechanisms that leads to the process of lipotoxicity by which reactive oxygen species (ROS), endoplasmic reticulum stress and hepatocellular injury are produced. In turn, liver cell injury activates the immune and apoptotic pathways, leading to cell death. This event is also one of the main drivers for the development of fibrosis and cirrhosis over time [64]. 

Hepatic steatosis can be caused by both aberrant lipid and glucose metabolism. One of leptin functions is to limit the storage of triglycerides in adipocytes and non-adipose tissues including the liver, thereby preventing lipotoxicity. Under normoleptinemia conditions, leptin exerts an anti-steatotic effect and improves insulin sensitivity by suppressing hepatic glucose production and lipogenesis [50,65]. This explains the improvement or prevention of hepatic steatosis development in ob/ob mice, linked to leptin administration [66]. Similarly, the anti-steatosis action of leptin has been observed in non-obese mice with uncontrolled type 1 diabetes mellitus (T1DM), in which such treatment induces a significant reduction of lipogenic and cholesterogenic transcription factors and decreases the lipids located in plasma and different tissues [67]. In this regard, one anti-steatotic mechanism carried out by leptin is to regulate components of the lipid synthesis in the liver, such as the transcription factor carbohydrate responsive element binding protein (ChREBP) [68].

Leptin has also been suggested to have a synergistic effect when used together with insulin, probably inhibiting the production of very low-density lipoproteins (VLDL) [10,67,69]. According to this, leptin has been shown to improve insulin resistance and hepatic steatosis in lypodystrophic mice [70]. Hackl et al. (2019) showed that brain leptin protects from ectopic lipid accumulation and could be a therapeutic strategy to improve obesity-related steatosis [71]. Moreover, this disease has been shown to alleviate upregulating leptin levels by using metformin [72] and through leptin signaling pathways by using a modification of Samjunghwan, an herbal formula used in traditional Korean medicine [73]. 

By contrast, high leptin levels have also been associated with hepatic steatosis and NAFLD pathogenesis since a high percentage of NAFLD patients have been observed to suffer obesity, which is closely related with hyperleptinemia [47,50,74]. The failure of elevated leptin levels to correct hepatic steatosis lies in the generation of a state of resistance to this hormone. Several mechanisms, including phosphorylation of Tyr985 in Ob-Rb and increased expression of SOCS-3, attenuate leptin signaling and promote a cellular resistance to leptin in obesity, which predominantly take place in the arcuate nucleus [75]. The severity of hepatic steatosis correlates with leptin levels, especially in patients with high BMI. In the case of lean patients with NAFLD, there are a number of genetic factors that seem to contribute to the development of steatosis rather than leptinemia, and these are hypobetalipoproteinemia and some metabolic disorders such as cystic fibrosis or celiac disease [76]. In addition, leptin has been reflected to have a pathogenic role in hepatic insulin resistance and/or a failure of the antisteatotic actions [39]. Cernea et al. (2018) observed an increased prevalence of NAFLD steatosis in T2DM patients [37]. Along the same lines, Pavlidis et al. (2011) showed that steatosis grade at baseline was significantly greater as leptin concentrations increased in chronic hepatitis C patients [77] and Eshraghian et al. (2020) demonstrated for the first time that alterations in adiponectin, leptin and insulin resistance were correlated with hepatic steatosis in liver transplant recipients [78].

### 3.2. Leptin, Non-Alcoholic Steatohepatitis (NASH), and Fibrosis

In NAFLD, most patients have simple steatosis, but those with NASH can advance to the next step of the disease, which is fibrosis. The mechanisms of progression from simple steatosis to NASH are not entirely clear, but some factors are known to be involved in the process [79], including an inflammation caused by the incomplete oxidation of hepatic accumulated lipids, which generates toxic metabolites and produces apoptosis of hepatocytes, thus activating inflammatory cells [80]. If inflammation becomes chronic, then fibrosis will be developed [81]. Related to this, leptin could promote NAFLD by playing its well-known role in the inflammatory process [17].

Advanced fibrosis implies an increased risk for developing other NAFLD-related complications, such as cirrhosis and HCC. For that reason, an early diagnosis of patients with advanced fibrosis is crucial [82]. In this sense, leptin has been shown to be a contributing factor in fibrogenesis [60]. Rotundo et al. (2018) showed that leptin levels were simultaneously increased with the degree of liver fibrosis, especially in patients with a high BMI, while their lean counterparts had lower rates of fibrosis and inflammation [76]. Some studies have reported that Ob-R on Kupffer cells (KC) and sinusoidal endothelial cells increases the expression of matrix remodeling enzymes, which induce the fibrosis cascade in hepatic stellate cells (HSC). Specifically, in KC leptin upregulates the expression of TGF- β, which is likely to contribute to HSC activation via paracrine signaling [18,76,83]. 

Activated HSC also contribute to increase inflammation and liver fibrosis by releasing TGF-β1, angiopoietin-1, VEGF (vascular endothelial growth factor), and collagen-I. In addition, HSC appear to produce leptin, and have also been proposed to express Ob-Rb, which establishes a vicious cycle by stimulating proliferation and preventing apoptosis of HSC and thus affecting hepatic inflammation and fibrosis [18]. KC can be activated by leptin via peroxynitrite-mediated oxidative stress [84], which promotes CD8^+^CD57^+^ T cells, found in NASH progression [85]. Also, prolonged hyperleptinemia may result in HSC, KC, and sinusoidal cell activation, that could trigger both the proinflammatory and profibrogenic cascade [50].

### 3.3. Leptin and Liver Cirrhosis 

According to several studies with small sample sizes, progression from NASH to liver cirrhosis can occur in up to 25% of patients. This high disease burden has led to an increase in the number of NASH-related transplants, possibly becoming in the leading cause of liver transplantation worldwide in coming decades, displacing the hepatitis C virus [86]. Up to now, leptin concentration in patients with NAFLD-related cirrhosis has not been studied. However, leptin is known to induce VEGF on HSC, contributing to the irreversibility of cirrhosis and, potentially, to NASH progression [87].

There are references in other types of liver cirrhosis about leptin, in which this hormone has been demonstrated to be in both high [88] and low [40] levels. Even leptin has also been found to be uncorrelated with the existence of cirrhosis in alcoholic liver disease [41]. Interestingly, Ockenga et al. (2007) analyzed in vivo hepatic substrate and leptin metabolism in 40 patients with liver cirrhosis and 31 healthy controls, showing that patients had bound leptin and soluble leptin receptor levels significantly increased when compared with controls, without changes in free leptin, suggesting a different role for those components in both metabolic and inflammatory processes in cirrhotic patients [42].

### 3.4. Leptin and Hepatocellular Carcinoma

Obesity and T2DM are cancer promoters and, in coexistence with NAFLD, the aggressive potential can be underestimated. HCC is the neoplasm most closely related to obesity in men. In this regard, HCC incidence increased by 3% per year in the last decade, unlike other malignancies also associated with obesity, such as breast or colon cancer, whose incidences remained stable or decreased. In part, this fact may be explained by the increase in the prevalence of NASH [89]. NAFLD patients have been shown to develop HCC in both early and late stages of the disease, being more common in the latter, providing evidence for a potential association between NAFLD and HCC [90]. The mechanisms of HCC development in a cirrhotic liver include destruction of hepatocytes due to chronic injury, and their subsequent regeneration and compensatory cyclic proliferation. NAFLD patients usually present insulin resistance which, together with hepatic steatosis and chronic low-grade inflammation, favors the creation of an ideal environment for tumor development and growth [91].

In HCC there are some established risk factors, including chronic hepatitis B, chronic hepatitis C, alcohol consumption, and NAFLD, all of them potentially linked to leptin [92]. As with NAFLD-related cirrhosis, there no clinical studies that analyze the role of leptin in NAFLD-related HCC. However, the procarcinogenic role of leptin in HCC patients seems clear. Additionally, high leptin levels alone are also considered to increase the risk of HCC [93]. In fact, in vitro studies suggest that this hormone is increased during the proliferation, migration, and invasiveness of HCC cells through activation of PI3K/AKT signaling pathways, mainly in obese patients [44] and have been demonstrated to take part in the angiogenesis process [94], as well as both JAK2/STAT and ERK pathways [95]. In line with this, a lack of leptin action has been shown to reduce the angiogenic process in experimental steatohepatitis [96]. Leptin also upregulates the expression of VEGF by oxygen-independent activation of hypoxia-inducible factor 1alpha (HF1α) in HSC [97]. Moreover, the analysis of circulating leptin levels has been found to be increased in both cirrhotic and non-cirrhotic patients regardless of the previous pathology [98], including NASH [43]. In this regard, more studies have also reported the role of leptin and Ob-R as a critical regulator in HCC development and progression [94,99,100].

However, Elinav et al. (2006) suggested a beneficial role of leptin in HCC murine models since this hormone decreased tumor size and improved survival [101]. In the same year, similar conclusions were drawn by analyzing both leptin and Ob-Rb in HCC patients [102,103]. Despite this, there is sufficient evidence to suggest the critical role of leptin in liver carcinogenesis, that may also be potentially fostered by NAFLD progression.

## 4. Concluding Remarks

NAFLD is a worldwide health problem due to its increasing prevalence, so the research on its diagnosis, follow-up, and subsequent treatment has become essential. Moreover, NAFLD requires a multidisciplinary approach given its high risk of cardiovascular morbidity and mortality. In this sense, there is an urgent need for non-invasive diagnostic methods to replace liver biopsy, so that early diagnosis and treatment monitoring is possible in a large part of the population. Leptin, due to its direct relationship with body fat levels and insulin resistance, has been shown to be an independent predictor of the presence or development of NAFLD. This adipokine has been shown to have antisteatotic effects, although it has also been associated with hepatic steatosis and may promote more advanced stages of NAFLD that include NASH and liver fibrosis. The role of leptin in both NAFLD-related cirrhosis and HCC has never been studied. Its functions in other liver cirrhosis remains controversial. However, there is much evidence to establish the protumoral role of this hormone in HCC derived from other liver diseases.

Treatment with leptin has proven to be effective in patients with congenital leptin deficiency; however, its use in the rest of the affected subjects remains controversial, which highlights the importance of continuing the line of research on the development of leptin analogues that conserve the antisteatotic effect and lack proinflammatory and profibrogenic action, as well as leptin sensitizers, or their synergistic effect when associated with different drugs. While further observational studies and large clinical trials with long-term follow-up are needed to fully evaluate the efficiency of the use of this adipokine, leptin could be used as an interesting biomarker in the diagnosis and follow-up of NAFLD, including the combination of leptin level measurement together with metabolic analyses, lipid profile, and glucose levels.

## Figures and Tables

**Table 1:** Overview about reviewed research articles in human non-alcoholic fatty liver disease (NAFLD) spectrum.

Author (Year)	Country	No Patients	Conclusions
Jacobs et al. (2011) [29]	Netherlands	434	Insulin resistance (IR) mediated between 75–80% of the association of the metabolic syndrome with alanine aminotransferase, as well as suggesting that IR, adipose tissue inflammation and endothelial dysfunction may contribute to NAFLD progression.
Hossain et al. (2015) [36]	Bangladesh	110	IR was independently associated with serum leptin levels irrespective of adiposity and glycemic status in male prediabetic subjects. In addition, serum leptin was increased in the female patients, accompanied by pancreatic beta cell dysfunction and IR. However, their relationship with NAFLD was not affected by the degree of adiposity.
Cernea et al. (2018) [37]	Romania	159	Hepatic steatosis was positively correlated with serum leptin and leptin resistance, and negatively with serum Ob-R. Leptin/Ob-R, and leptin resistance did not made a significant contribution to hepatic fibrosis.
Angulo et al. (2004) [38]	U.S.A.	88	There was no association between serum leptin and hepatic fibrosis. However, there was a correlation between leptin with more advanced NAFLD-related liver fibrosis.
Chitturi et al. (2002) [39]	Australia	36 patients and 47 controls	Hyperleptinemia in NASH was correlated with some factors (e.g., age and extent of hepatic steatosis), but not with inflammation or fibrotic severity.
Ataseven et al. (2006) [40]	Turkey	45 patients (23 cirrhosis + 22 HCC) and 25 controls	In cirrhosis and HCC patients there was a decrease of serum leptin levels due to, at least partly, the presence of nutritional and metabolic abnormalities, including malnutrition, and high ghrelin levels.
Naveau et al. (2006) [41]	France	209	Serum leptin was independently correlated with steatosis and may play an important role in severity of fibrosis.
Ockenga et al. (2007) [42]	Germany	40 liver cirrhosis + 31 controls	Patients had bound leptin and soluble leptin receptor levels significantly increased compared with controls, without changes in free leptin.
Ertle et al. (2011) [43]	Germany	162	NAFLD/NASH posed a risk factor for HCC, even in the absence of cirrhosis.

## Data Availability

Not applicable.
